# Pro- and Anti-Inflammatory Responses in Severe COVID-19-Induced Acute Respiratory Distress Syndrome—An Observational Pilot Study

**DOI:** 10.3389/fimmu.2020.581338

**Published:** 2020-10-06

**Authors:** Quirin Notz, Marc Schmalzing, Florian Wedekink, Tobias Schlesinger, Michael Gernert, Johannes Herrmann, Lena Sorger, Dirk Weismann, Benedikt Schmid, Magdalena Sitter, Nicolas Schlegel, Peter Kranke, Jörg Wischhusen, Patrick Meybohm, Christopher Lotz

**Affiliations:** ^1^Department of Anesthesiology and Intensive Care Medicine, University Hospital Würzburg, Würzburg, Germany; ^2^Department of Medicine II, Rheumatology and Clinical Immunology, University Hospital Würzburg, Würzburg, Germany; ^3^Department of Gynecology, Section for Experimental Tumor Immunology, University Hospital Würzburg, Würzburg, Germany; ^4^Department of Internal Medicine I, University Hospital Würzburg, Würzburg, Germany; ^5^Department of General, Visceral, Vascular and Pediatric Surgery (Surgery I), University Hospital Würzburg, Würzburg, Germany

**Keywords:** Coronavirus Disease 2019, acute respiratory distress syndrome, Severe Acute Respiratory Syndrome Coronavirus 2, cytokines, inflammation, growth differentiation factor 15, immune response

## Abstract

**Objectives:**

The severity of Coronavirus Disease 2019 (COVID-19) is largely determined by the immune response. First studies indicate altered lymphocyte counts and function. However, interactions of pro- and anti-inflammatory mechanisms remain elusive. In the current study we characterized the immune responses in patients suffering from severe COVID-19-induced acute respiratory distress syndrome (ARDS).

**Methods:**

This was a single-center retrospective study in patients admitted to the intensive care unit (ICU) with confirmed COVID-19 between March 14^th^ and May 28^th^ 2020 (n = 39). Longitudinal data were collected within routine clinical care, including flow-cytometry of lymphocyte subsets, cytokine analysis and growth differentiation factor 15 (GDF-15). Antibody responses against the receptor binding domain (RBD) of Severe Acute Respiratory Syndrome Coronavirus 2 (SARS-CoV-2) Spike protein were analyzed.

**Results:**

All patients suffered from severe ARDS, 30.8% died. Interleukin (IL)-6 was massively elevated at every time-point. The anti-inflammatory cytokine IL-10 was concomitantly upregulated with IL-6. The cellular response was characterized by lymphocytopenia with low counts of CD8+ T cells, natural killer (NK) and naïve T helper cells. CD8+ T and NK cells recovered after 8 to 14 days. The B cell system was largely unimpeded. This coincided with a slight increase in anti-SARS-CoV-2-Spike-RBD immunoglobulin (Ig) G and a decrease in anti-SARS-CoV-2-Spike-RBD IgM. GDF-15 levels were elevated throughout ICU treatment.

**Conclusions:**

Massively elevated levels of IL-6 and a delayed cytotoxic immune defense characterized severe COVID-19-induced ARDS. The B cell response and antibody production were largely unimpeded. No obvious imbalance of pro- and anti-inflammatory mechanisms was observed, with elevated GDF-15 levels suggesting increased tissue resilience.

## Introduction

Critical Coronavirus Disease 2019 (COVID-19) leads to an acute respiratory distress syndrome (ARDS), requiring intensive care unit (ICU) support and long recovery times. The immune response to Severe Acute Respiratory Syndrome Coronavirus 2 (SARS-CoV-2) critically determines the clinical course and severity of COVID-19. First studies indicate that lymphocytopenia is a hallmark of COVID-19, correlating with disease severity (1–3). Moreover, high Interleukin (IL)-6 was identified as a predictor of mortality (4, 5). Virus elimination and recovery depend on pro-inflammatory signals alerting the immune system. Innate immune sensing, cytokine synthesis, myeloid cell, and leukocyte activation with a solid T cell response are pivotal to quickly locate and combat the virus. Cytotoxic cells, including tissue-resident memory CD8+ T cells, provide the first line of defense and reduce viral burden. B cells present antigen to CD4+ T cells which in turn provide helper signals to produce specific antibodies against SARS-CoV-2 a few days later.

An effective immune response cannot be replaced by any therapeutic intervention. Nevertheless, inflammation is a double-edged sword. Uncontrolled hyperinflammation results in cytokine release syndrome (CRS) leading to tissue damage, apoptosis of immune cells, and impaired cytotoxic function. In a positive feedback-loop CRS is amplified by the key cytokines IL-6, interferon gamma (IFN*γ*), and tumor necrosis factor alpha (TNFα). Anti-inflammatory cytokines such as IL-10 control the pro-inflammatory response. Ideally pro- and anti-inflammatory mechanisms should be balanced. However, in clinical practice this state is nearly impossible to decipher as *e.g.* elevated levels of IL-6 and low lymphocyte counts (6, 7) do not exclusively discriminate balanced from unbalanced inflammation.

Another feature besides viral infectivity and inflammation is the host response and tissue tolerance. An emerging modulator of immune responses (8) and mediator of inflammation tissue tolerance is the multi-functional anti-inflammatory cytokine growth and differentiation factor 15 (GDF-15) (9), which physiologically promotes immunotolerance during pregnancy. In mouse models GDF-15 protects the kidney, liver, and other organs from adverse consequences of bacterial and viral sepsis (9, 10). After ischemia–reperfusion injury, GDF-15 protects the infarcted myocardium by limiting the influx of inflammatory cells (10). GDF-15 may, however, also attenuate antiviral immune responses. In mice infected with human rhinovirus GDF-15 supports viral replication, thereby promoting virus-induced lung inflammation (11). Thus, GDF-15 has the potential to significantly affect the outcome of a viral infection.

In the current study we investigated the immune response in patients suffering from severe COVID-19-induced ARDS. We are the first to exclusively characterize a COVID-19 ICU patient population with an expected mortality of >46% (12). In order to provide a comprehensive characterization, longitudinal measurements of extensive T and B cell subsets, natural killer (NK) cells as well as pro- and anti-inflammatory cytokines were conducted. Levels of anti-SARS-CoV-2-Spike-receptor binding domain (RBD) serum antibodies were determined as part of the humoral immune response. Together with other data on pro- and anti-inflammatory immune regulators we also investigated GDF-15 serum levels as this cytokine may be predictive for tissue tolerance during severe inflammation.

## Patients, Materials, and Methods

### Study Design and Patients

This is a retrospective single-center cohort study adhering to the STROBE-Guidelines (13). All patients admitted to the ICU at the University Hospital of Würzburg with a confirmed COVID-19 diagnosis between March 14^th^ and May 28^th^ 2020 were evaluated for study eligibility (n = 39). Of those 13 patients retrospectively fulfilled the study entry criteria (*i.e.* presence of moderate or severe ARDS and advanced immunologic diagnostics as described below). The University Hospital of Würzburg provides tertiary care and is a referral center for adult extracorporeal membrane oxygenation (ECMO) with a radius of more than 100 km. Hence, all COVID-19 patients in the current study were transferred from other regional hospitals *via* the German “ARDS network”. A SARS-CoV-2 infection was confirmed *via* standardized real-time reverse transcriptase polymerase chain reaction (RT-PCR) testing both tracheal aspirate and pharyngeal swab upon admission (14). Data were collected *via* retrospective chart review within standard care. Due to the severity of COVID-19 in our ICU patients, routine laboratory analyses were extended by a broad spectrum of inflammation mediators, lymphocyte subsets, and cytokine panels. Specific treatment protocols were not defined. Due to sole chart review, the institutional board of the University of Würzburg waived the need for ethic approval (63/20-kr, 25.03.2020 and 20200528 01, 05.06.2020). Informed consent from patients was not necessary according to local legislation (Bayerisches Krankenhausgesetz, Art. 24, Abs 4). Samples from internal healthy controls (HC) were obtained from volunteers working at the University of Würzburg with a median age of 29 (27–45) years.

### Data Collection

Clinical data were continuously recorded and obtained from the patient data management system (COPRA6 RM1.0, COPRA System GmbH, Berlin, Germany). The software includes a calculator to assess the sequential organ failure assessment (SOFA) score, which is recommended for the evaluation of disease severity in all septic and critically ill patients on a daily basis according to The Third International Consensus Definitions for Sepsis and Septic Shock (Sepsis-3) (15). Furthermore the acute physiology and chronic health evaluation (APACHE) IV score was regularly calculated using an online tool (16). APACHE IV has been validated in a huge multicenter study as a predictor of in-hospital mortality (17). Severity of ARDS was defined according to the Berlin definition (18).

Prior medical history was evaluated *via* written records. Blood test results were analyzed from routine samples drawn between days 1 and 4 (“admission”), 5 and 8, 11 and 14, 18 and 21 and 28 and 31. Routine laboratory parameters include complete blood counts, markers of inflammation, immunoglobulin (Ig) A, M and G levels as well as liver and renal function. Arterial blood gas (ABG) samples were recorded daily. Immunoglobulin levels are only depicted in patients (n = 10) who did not receive immunoglobulin during ICU treatment or Rituximab (RTX) prior to ICU admission (**Table Supp 1**).

EDTA anticoagulated whole blood staining was used for fluorescence-activated cell sorting. Lymphocyte subsets were analyzed with a Navios cytometer (Beckman Coulter, Krefeld, Germany). A minimum of 3,000 events within each lymphocyte gate was collected. In order to identify different T cell subsets the following anti-human antibodies were used in one staining: anti-CD45-Krome-Orange, anti-CD14-APCA700, anti-CD3-FITC, anti-CD4-APC, anti-CD8-ECD, anti-CCR7-PC7, anti-CD45RA-PB, anti-TCR*γ*/*δ*-PC5.5, anti-CD56/CD16-APC A750 (each Beckman Coulter, Krefeld, Germany). First, lymphocytes were gated using forward *versus* side scatter and CD45 alongside exclusion of monocytes. CD45 high CD3+ events were then defined as T cells (CD3+) and further differentiated into T helper cells (CD3+ CD4+) and CD8+ T cells (CD8+ CD3+). CCR7+ CD45RA+ cells within the T helper cell population indicated naïve T helper cells (CD3+ CD4+ CCR7+ CD45RA+). Natural killer T (NKT)-like cells (CD3+ CD56/CD16+) were differentiated from NK cells (CD3− CD56/CD16+). Expression of the *γδ* T cell receptor defined *γδ* T cells (CD3+ *γδ*+). B cell subsets were analyzed with the following anti-human antibodies in one staining: anti-CD45-Krome-Orange, anti-CD19-PC7, anti-CD38-PC5.5, anti-CD27-ECD, anti-CD20-APC750 (each Beckman Coulter, Krefeld, Germany), anti-CD10-PE, anti- IgD-FITC (each BD Biosciences, San Jose, USA), and anti-CD21-PB (Exbio, Prague, Czech Republic). First, lymphocytes were gated using forward *versus* side scatter and CD45. CD45 high CD19+ events were identified as B cells (CD19+). A high or intermediate staining for CD10 and concomitant CD38 positivity indicated transitional B cells (CD19+ CD10+ CD38+). Next, staining of CD27 and IgD allowed us to differentiate naïve B cells (CD19+ CD27− IgD+), pre-switch memory B cells (CD19+ CD27+ IgD+), post-switch memory B cells (CD19+ CD27+ IgD-) and double negative B cells (CD19+ CD27− IgD−). High expression of CD38 and negative or low staining for CD20 in CD27+ IgD− cells characterized circulating plasmablasts (CD19+ CD38+ CD27+ IgD−). CD21^low^ B cells (CD19+ CD38^dim^ CD21^dim^) were also analyzed. B cell subsets from RTX-patients (n = 2) were excluded from the analysis.

T cell subsets and NK cells are presented as percentage of the total lymphocyte count, with exception of naïve T helper cells, which refer to the total numbers of T helper cells. B cell subsets are displayed as percentage of total B cells. Reference values are based on previous publications (19–24).

Cytokine concentrations of human IL-1β, IL-2, IL-7, IL-10, IL-17a, interferon gamma-induced protein 10 (CXCL10, also known as IP-10), IFN*γ*, TNFα, and granulocyte macrophage colony-stimulating factor (GM-CSF) were analyzed using a commercially available multi-analyte immunoassay and Luminex^®^ bead technology with reagent kits (Merck Millipore, Burlington, USA) according to the manufacturer’s instructions. The specific cytokine panel was chosen in order to display features of Th1-, Th2-, Th17-immune responses and macrophage activation. Individual patient sera were measured in doublets. Due to laboratory safety restrictions we could only measure SARS-CoV-2-ribonucleic acid (RNA) negative serum blood samples, which required the exclusion of two patients from the analysis. Cytokine analysis was primarily conducted in patients without hemadsorption therapy. However, two samples (one on admission, one on days 5–8) were collected during hemadsorption therapy (CytoSorb^®^, CytoSorbents™, New Jersey, USA). Porous beads within the filter absorb cytokines between 10 and 55 kD, whereas low cytokine concentrations are not altered (25). Cytokine reference values are based on pooled internal healthy controls. The minimal detectable concentration for the different cytokines is indicated in brackets: IL-1*β* (0.37 pg/ml), IL-2 (0.32 pg/ml), IL-7 (0.029 pg/ml), IL-10 (0.15 pg/ml), IL-17a (0.3 pg/ml), CXCL10 (1.23 pg/ml), IFN*γ* (0.019 pg/ml), TNFα (1.41 pg/ml), GM-CSF (0.72 pg/ml).

GDF-15 levels were determined with an enzyme-linked immunosorbent assay (ELISA). A modified version of the R&D DuoSet kit (R&D Systems, Minneapolis, USA) was used with an in-house-developed anti-GDF-15 antibody (patent: EP3122775A1) for capture. Serum of COVID-19 patients was diluted 1:500; serum of HC was diluted 1:10. The assay can reliably quantify GDF-15 levels ≥0.1 ng/ml. Anti-SARS-CoV-2-Spike-RBD detection (ELISA) was performed as previously described with small adaptations to local needs (26, 27). RBD was recombinantly expressed in Expi293F HEK cells using the RBD_6His expression plasmid (26, 27). Serum samples were diluted 1:50. For detection, cross-adsorbed isotype specific horseradish peroxidase-conjugated anti-human IgG (Thermo Scientific, Waltham, USA), anti-human IgM (YO Proteins, Rönninge, Sweden), and anti-human IgA (International Limited, New Delhi, India) was used at a 1:10,000 dilution. Based on standard curves the assay could quantify anti-SARS-CoV-2-Spike-RBD IgG levels ≥123 ng/ml and Anti-SARS-CoV-2-Spike-RBD IgM and IgA levels ≥4 ng/ml. ELISA readout was performed on a Tecan sunrise (Tecan, Männedorf, Switzerland) at 450 nm, corrected at 620 nm. Origin (OriginLab Corporation, Northhampton, USA) was used to fit the standard curves.

### Statistical Analysis

Median and interquartile ranges (IQR, 25–75%) were calculated for all variables, as normality of the data could not be assumed. To compare differences between HC and COVID-19 patients across continuous variables the Mann–Whitney rank-sum test was used. The Bonferroni correction for multiple testing was applied and subsequent differences were considered significant with p < 0.01. To analyze changes over multiple time-points within the COVID-19 cohort a mixed-effect model for repeated measures accounted for missing values. We used the Geisser–Greenhouse correction and compared the different time points with Tukey’s multiple comparisons test. Differences were considered significant with an adjusted p < 0.05. Associations between different variables were correlated according to Spearman. All p-values and Spearman’s correlation coefficients (r_s_) are reported in full. In figures, significant differences are indicated with bars and p-values connecting either HC and COVID-19 patients (Mann–Whitney test) or different time points within the COVID-19 cohort (mixed-effect model). Data preparation was done with Microsoft Office^®^ 365 ProPlus (Microsoft™, Redmond, USA) and GraphPad Prism^®^ Version 8.4.2 (GraphPad Software™, San Diego, USA).

## Results

### Demographics

Patients had a median age of 58 (54–66) years, 76.9% were male and 23.1% female. Hospital transfer to Würzburg in median took place on day 11 (10–24) after symptom onset (**Figure Supp 1**). All patients were already mechanically ventilated on admission, the median SOFA score was 15 (14–15), the APACHE IV score was 108 (104–114), and the median pO_2_/FiO_2_ ratio was 96 mmHg (72–178). We found significant correlations between SOFA and APACHE IV for days 1–4 (p = 0.0446), days 5–8 (p = 0.0063), days 11–14 (p = 0.0010) and days 18–21 (p = 0.0085). Six patients (46.2%) had moderate, seven (53.8%) severe ARDS on admission. All patients suffered from severe ARDS during the course of ICU therapy with the lowest median pO_2_/FiO_2_ ratio being 60 (53–69). Veno-venous extracorporeal membrane oxygenation (vvECMO) was required in eight patients (61.5%), renal replacement therapy in eleven (84.6%). In eight patients renal replacement therapy was at least once combined with a hemadsorption device. Eight patients received glucocorticoids. Immunoglobulin was administered in three and tocilizumab in one case (**Table Supp 1**). Patients stayed in intensive care for a median of 34 days (18–43). SOFA peaked at a median of 17 (17–18); APACHE IV reached 114 (106–124) respectively. Nine patients survived to ICU discharge (69.2%), four patients died from multi-organ failure after a median length of stay of 14 days (8.3–22.8; **Table 1**). Coagulation parameters are shown in **Table 2**. It is important to note that all patients received heparin with a target activated partial thromboplastin time (aPTT) of 40–50 s, as thrombotic microangiopathy was early on suggested as a hallmark of COVID-19 (28).

**Table 1:** Demographics and clinical course.

Characteristics	Patients (n = 13)
Female, No. patients (%)	3 (23.1)
Male, No. patients (%)	10 (76.9)
Age, years (median, IQR)	58 (54–66)
Comorbidities	
Body mass index, kg/m^2^ (median, IQR)	29.1 (25.7–30.9)
Respiratory comorbidity, No. patients (%)	3 (23.1)
Diabetes mellitus type II, No. patients (%)	3 (23.1)
Malignancy, No. patients (%)	3 (23.1)
Chemotherapy and stem cell transplant <6 months, No. patients (%)	1 (7.7)
Rheumatoid arthritis, No. patients (%)	2 (15.4)
Clinical characteristics on admission	
Heart rate, beats per minute (median, IQR)	100 (82–105)
Temperature, °C (median, IQR)	36.9 (36.5–37.8)
PaO_2_/FiO_2_, mmHg (median, IQR)	96 (72–178)
SOFA score (median, IQR)	15 (14–15)
APACHE IV score (median, IQR)	108 (104–114)
Clinical characteristics during intensive care	
Duration of ICU stay, days (median, IQR)	34 (18–43)
Mechanical ventilation, days (median, IQR)	28 (10–34)
vvECMO, No. patients (%)	8 (61.5)
vvECMO, total hours (median, IQR)	336 (212–432)
Cessation of vvECMO, days after admission (median, IQR)	17 (13.3–23.8)
Renal replacement therapy, No. patients (%)	11 (84.6)
Highest SOFA score (median, IQR)	17 (17–18)
Highest APACHE IV score (median, IQR)	114 (106–124)
Minimal PaO_2_/FiO_2_, mmHg (median, IQR)	60 (53–69)
Highest PaCO_2_, ABG, mmHg (median, IQR)	77 (68.8–105)
Outcome	
Deceased patients, No. patients (%)	4 (30.8)
Survival upon ICU discharge, No. patients (%)	9 (69.2)

ABG, arterial blood gas; APACHE, acute physiology and chronic health evaluation; ICU, intensive care unit; IQR, interquartile range; No., number of patients; PaCO_2_, arterial partial pressure of carbon dioxide; PaO_2_/FiO_2_, ratio of arterial oxygen partial pressure and fraction of inspired oxygen (Horovitz index); SOFA, sequential organ failure assessment score; vvECMO, veno-venous extracorporeal membrane oxygenation.

**Table 2:** Coagulation parameters during intensive care.

Coagulation	Days 1–4	Days 5–8	Days 11–14	Days 18–21	Days 28–31
Median, IQR	n = 13	n = 13	n = 10	n = 10	n = 7
Platelets × 1,000/µl	196 (95–272)	169 (135–322)	191 (129–288)	172 (140–286)	216 (124–395)
aPTT, s	43.5 (40–49)	42.9 (40–51)	50.8 (50–55)	38.1 (37–59)	39 (34–45)
INR	1 (0.9–1.1)	1 (0.9–1)	1 (1–1.1)	1 (1–1.1)	0.9 (0.9–1.1)
D-dimers, mg/L	4.1 (3–8)	3 (3–8)	8.8 (5–16)^†^	10.3 (5–19)^†^	10 (8–12)^†^

aPTT, activated partial thromboplastin time; INR, international normalized ratio; IQR, interquartile range. ^†^Missing data for some patients.

### Lymphocyte Count

**Figure 1:**
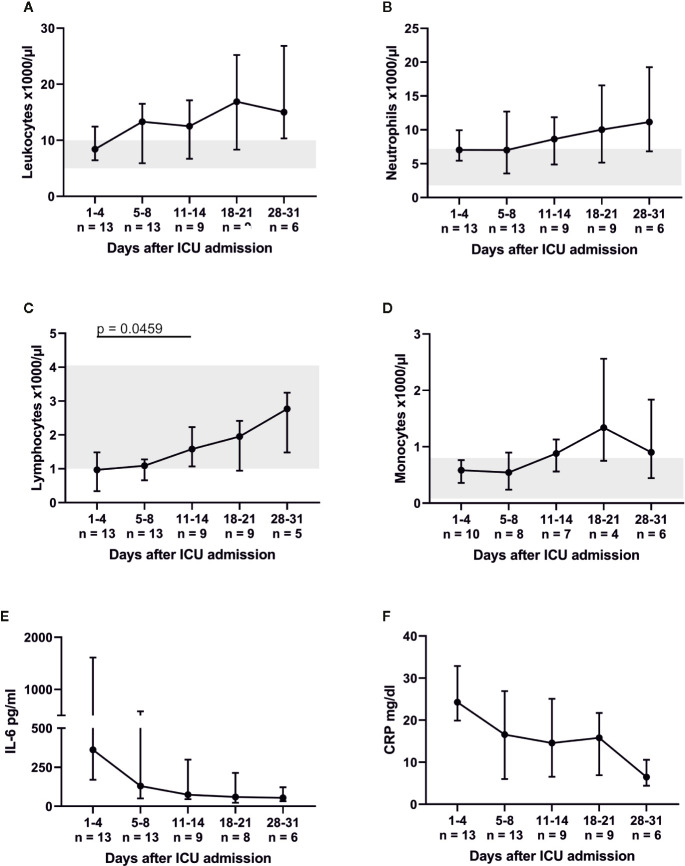
Severe COVID-19-induced ARDS was accompanied by lymphocytopenia, which recovered between admission and days 11–14. **(A**–**D)** indicate the total numbers of leukocytes as well as their respective subpopulations. While neutrophils and monocytes were within their respective reference range (marked gray area), lymphocyte counts were reduced on ICU admission. Concomitantly interleukin (IL)-6 (reference range 0–7 pg/ml) and C-reactive protein (CRP, reference range 0–0.5 mg/dl) were massively elevated **(E, F)**.

**Figure 2:**
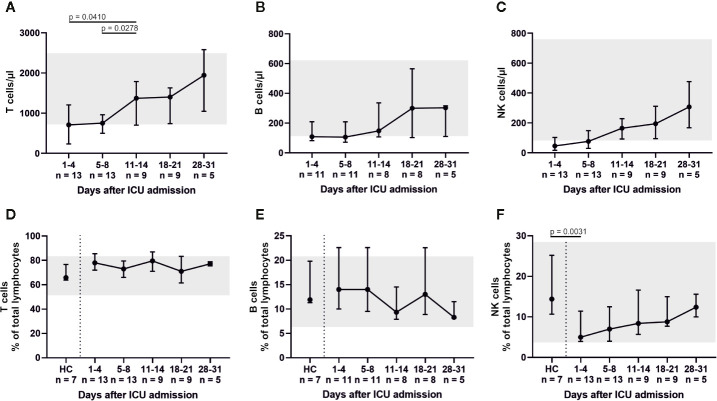
All distinct lymphocyte subsets (T cells, B cells, and NK cells) exhibited low cell counts on ICU admission. In particular, absolute and relative numbers of natural killer (NK) cells were reduced. These findings suggest an impaired or delayed cytotoxic reaction, as NK cell counts slowly increased over the course of intensive care. Absolute numbers are shown in **(A**–**C)**, while relative cell counts are displayed in **(D**–**F)**. HC, healthy controls.

Peripheral blood lymphocytes were below their reference range on admission. However, white blood cell count (WBC) and its subsets increased (almost tripled) over the course of the ICU treatment (**Figures 1A–D**). The lymphocytic recovery between admission and days 11–14 was statistically significant (p = 0.0459). Absolute numbers of CD3+ T, CD19+ B and CD3− CD56/CD16+ NK cells increased over time (**Figure 2**) with a significant difference of the T cell counts between admission and days 11–14 (p = 0.041) and between days 5–8 and days 11–14 (p = 0.0278). Relative proportions of T and B cells did not change, proportions of NK cells were significantly reduced on admission compared to HC (p = 0.0031) and slightly increased over the course of intensive care.

### T Cell Subset Analyses

**Figure 3:**
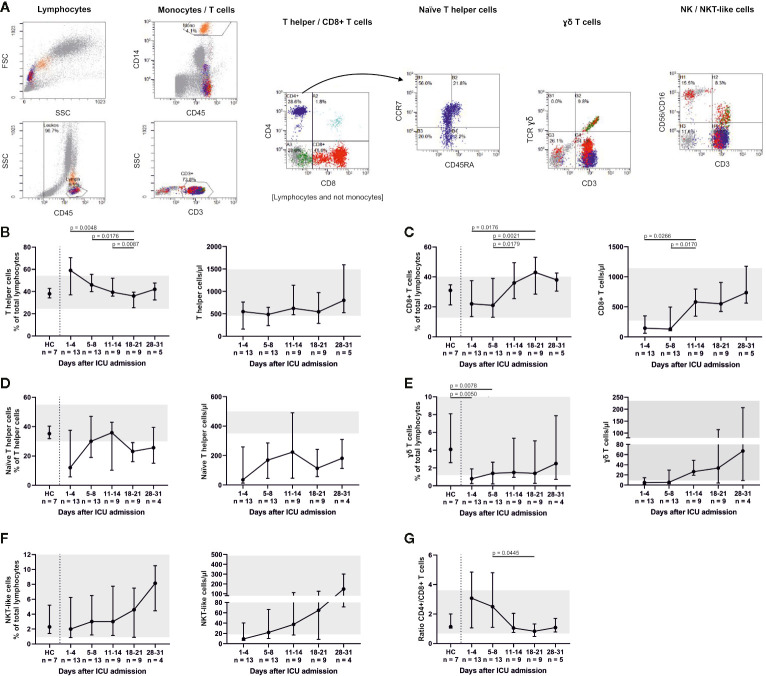
T cell subsets were differentiated by flow cytometry (representative gating, **A**) and demonstrated considerably reduced CD3+ CD8+ T cells and CD3+ CD4+ CCR7+ CD45RA+ naïve T helper cells. Both fractions slowly increased during treatment with a maximum between days 11 and 21. Absolute numbers of naïve T helper cells remained far below the reference range throughout ICU therapy **(B**–**D)**. CD3+ *γδ*+ T cells were also significantly reduced at admission and only partly replenished **(E)**, whereas CD3+ CD56/CD16+ natural killer T (NKT)-like cells increased towards the end of ICU treatment **(F)**. A declining CD4+/CD8+ ratio reflected these changes **(G)**. Overall, these findings suggest that severe COVID-19-induced ARDS was accompanied by a T cell response with a delayed cytotoxic reaction. Relative cell counts on the left of each pair of graphs are contrasted with absolute cell numbers on the right, respectively. FSC, forward scatter; SSC, side scatter; HC, healthy controls; TCR, T cell receptor.

We further analyzed the abundance of major T cell subsets (**Figure 3**). Percentages of CD3+ CD4+ T helper cells significantly decreased (admission to day 18–21, p = 0.0048; days 5–8 to days 18–21, p = 0.0176; days 11–14 to days 18–21, p = 0.0087) over time, while percentages (admission to days 18–21, p = 0.0176; days 5–8 to days 11–14, p = 0.0179; days 5–8 to days 18–21, p = 0.0021) and absolute numbers (admission to days 11–14, p = 0.0266; days 5–8 to days 11–14, p = 0.017) of CD3+ CD8+ T cells increased. This resulted in a declining CD4+/CD8+ ratio (days 5–8 to days 18–21, p = 0.0445). The lowest percentage of CD3+ CD4+ CCR7+ CD45RA+ naïve T helper cells was counted on admission with a median of 12% (5.7–37.5). Naïve T helper cells increased up to days 5–8 and days 11–14, but then again fell below the reference range of 30%. Absolute numbers of naïve T helper cells remained far below the reference range throughout ICU therapy. CD3+ *γδ*+ T cells were significantly reduced at admission and days 5–8 in comparison to HC (p = 0.005 and p = 0.0078) and replenished over the course of the ICU stay. CD3+ CD56/CD16+ NKT-like cells also increased.

### B Cell System and Anti-SARS-CoV-2 Antibody Response

**Figure 4:**
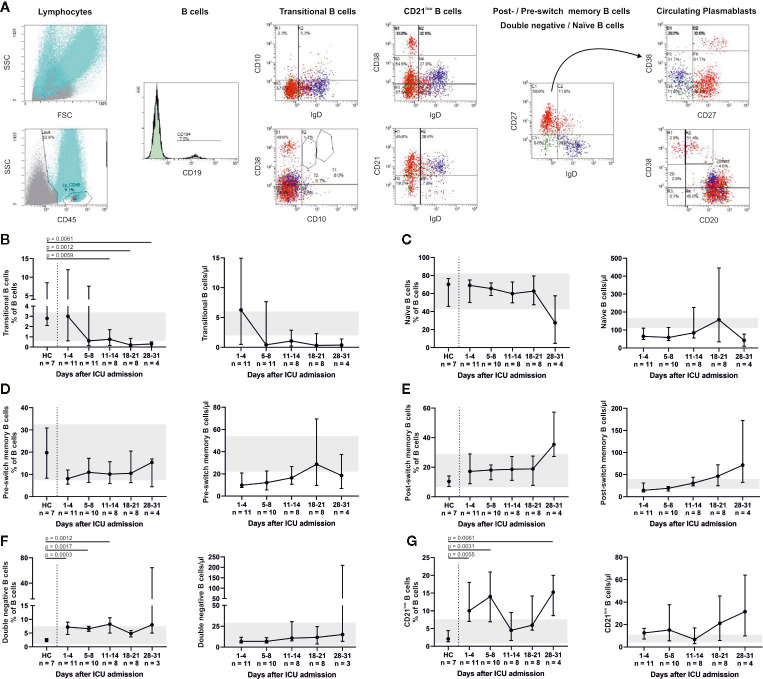
Flow cytometry indicated a robust B cell response throughout ICU treatment (representative gating, **A**). CD19+ CD10+ CD38+ transitional B cells decreased **(B)**, whereas the fraction of CD19+ CD27− IgD+ naïve B cells did not change. Absolute numbers of naïve B cells were below their reference range **(C)**. These findings indicate a high turnover of immature and undifferentiated cells during lymphocytic recovery. Accordingly, pre- (CD19+ CD27+ IgD+) **(D)** and consecutively post-switch memory B cells (CD19+ CD27+ IgD−) **(E)** increased over time. We also observed higher fractions of CD19+ CD27− IgD− double negative (DN) B cells **(F)** and CD19+ CD38^dim^ CD21^dim^ B cells **(G)** compared to healthy controls (HC). CD21^low^ B cells also partially exceeded the normal range. Relative cell counts on the left of each pair of graphs are contrasted with absolute cell numbers on the right, respectively. FSC, forward scatter; SSC, side scatter.

**Figure 5:**
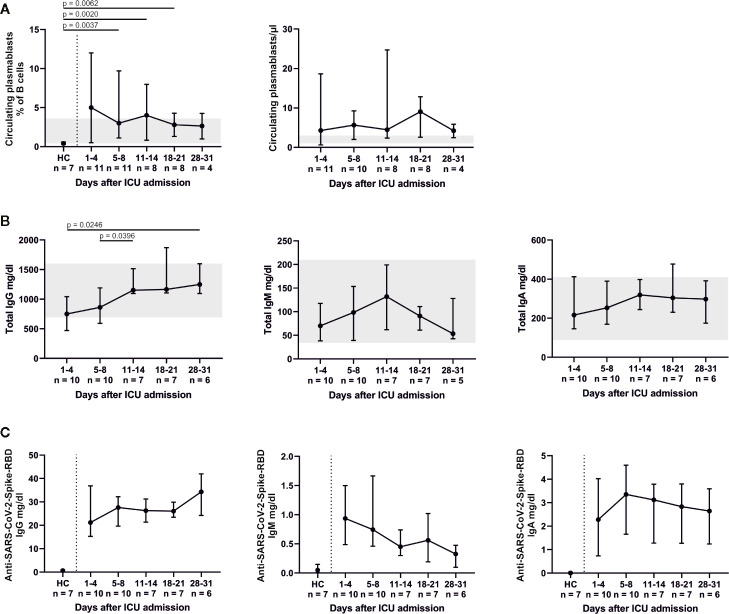
The B cell response led to an elevation in CD19+ CD38+ CD27+ IgD− circulating plasmablasts **(A)**. Total Immunoglobulin (Ig) levels plateaued or peaked after 11–14 days **(B)**. The increase of total IgG was mirrored by slightly rising levels of anti-SARS-CoV-2-Spike-receptor binding domain (RBD) IgG and decreasing levels of anti-SARS-CoV-2-Spike-RBD IgM. Anti-SARS-CoV-2-Spike-RBD IgA levels were fully pronounced on days 5–8. All anti-SARS-CoV-2-Spike-RBD serum antibodies were already present at the time of ICU admission **(C)**. HC, healthy controls.

Relative changes in the B cell system are shown in **Figure 4**. A significant decrease in CD19+ CD10+ CD38+ transitional B cells compared to HC was found for days 11–14 (p = 0.0059), days 18–21 (p = 0.0012) and days 28–31 (p = 0.0061). Percentages of CD19+ CD27− IgD+ naïve, CD19+ CD27+ IgD+ pre-switch memory and CD19+ CD27+ IgD− post-switch memory B cells remained relatively unaltered, while absolute numbers of post-switch memory B cells increased over time. We furthermore found a significant elevation of CD19+ CD27− IgD− double negative B cells (admission, p = 0.0003; days 5–8, p = 0.0017; days 11–14, p = 0.0012) and CD19+ CD38^dim^ CD21^dim^ B cells (admission, p = 0.0055; days 5–8, p = 0.0031; days 28–31, p = 0.0061) compared to HC. Also, CD19+ CD38+ CD27+ IgD− circulating plasmablasts were significantly increased at days 5–8 (p = 0.0037), days 11–14 (p = 0.002) and days 18–21 (p = 0.0062) in comparison to HC. This coincides with an overall increase of total IgG (admission to days 28–31, p = 0.0246; days 5–8 to days 11–14, p = 0.0396) as well as a slight, non-significant increase of anti-SARS-CoV-2-Spike-RBD IgG. Concomitantly anti-SARS-CoV-2-Spike-RBD IgM decreased and anti-SARS-CoV-2-Spike-RBD IgA remained relatively unaltered (**Figure 5**). In median 2.7% (2.3–2.8) of total IgG, 0.6% (0.6–0.8) of total IgM and 1% (0.9–1.1) of total IgA were specific for SARS-CoV-2-Spike-RBD.

### Cytokine Profile

**Figure 6:**
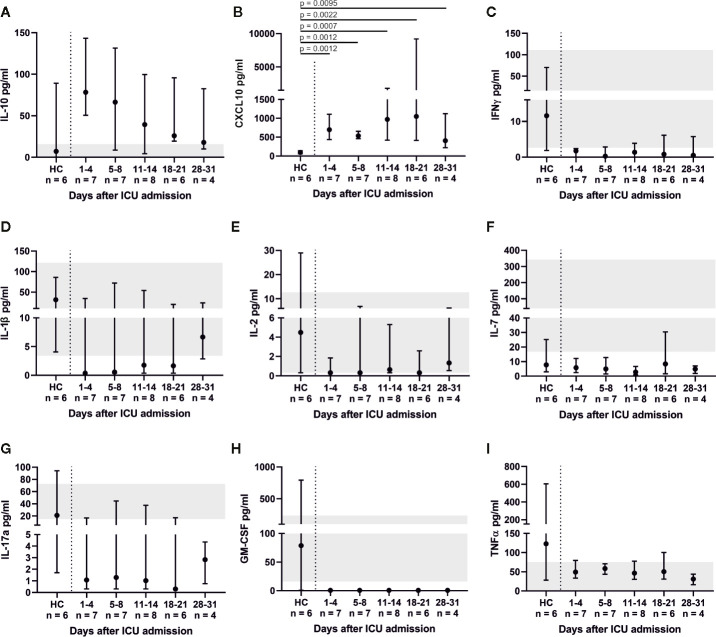
Cytokine profile of COVID-19 ARDS patients. Anti-inflammatory interleukin (IL-)10 dynamics were similar to IL-6 **(A)**. Interferon gamma (IFN*γ*), tumor necrosis factor alpha (TNFα), IL-1*β*, IL-2, IL-7, IL-17a and granulocyte macrophage colony-stimulating factor (GM-CSF) **(C**–**H)** mostly exhibited levels below their respective reference range. Interferon gamma induced protein 10 (CXCL10) was significantly elevated in comparison to healthy controls (HC, reference range 12.1–40.8 pg/ml) **(B)**.

**Figure 7:**
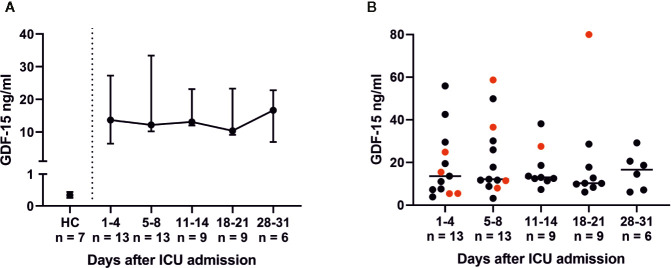
Growth differentiation factor 15 (GDF-15) serum levels in COVID-19 patients with acute respiratory distress syndrome (ARDS). GDF-15 levels were determined *via* an enzyme-linked immunoassay (ELISA) with an in-house-developed anti-GDF-15 antibody (patent: EP3122775A1). Serum levels of GDF-15 were elevated in all COVID-19 ARDS patients compared to healthy controls (HC) **(A)**. Non-survivors had rising GDF-15 levels (red dots), while survivors had tendentiously decreasing levels (black dots) of GDF-15 **(B)**. GDF-15 upregulation is likely part of an adaptive response in order to restore a disturbed balance between pro- and anti-inflammatory cytokines.

Throughout ICU treatment we found massively elevated levels of IL-6 along with high C-reactive protein (CRP) levels (**Figures 1E, F**). There was also a significant inverse correlation between lymphocyte count and IL-6 levels on admission (p = 0.0025; r_s_ = −0.7802). This pro-inflammatory signature may have been partly counter-balanced by a dynamic increase in IL-10. Elevated levels of IL-6, CRP, and IL-10 on admission decreased over time and approximated HC. CXCL10 was significantly elevated at every point of time compared to its reference range and HC (admission and days 5–8, p = 0.0012; days 11–14, p = 0.0007; days 18–21, p = 0.0022; days 28–31, p = 0.0095). Interestingly, IFN*γ*, IL-1*β*, IL2, IL-7, IL-17a and GM-CSF levels were below their respective reference range. TNFα was within its reference range and did not change over time (**Figure 6**). Additionally, we measured GDF-15, which displayed considerably higher levels in all COVID-19 patients (**Figure 7**). We refrained from a statistical comparison between HC and COVID-19 patients with regard to GDF-15. A different dilution had to be used in HC, as these would otherwise have had values below the detection limit. GDF-15 levels did not correlate with the patient’s body mass index or age.

## Discussion

In the current study we characterized the cellular and humoral immune response during ICU treatment of COVID-19. All patients suffered from severe ARDS during the course of therapy. 61.5% of the patients required vvECMO support. Our data show massively elevated levels of IL-6 and a cellular response characterized by lymphocytopenia and delayed cytotoxic immune defense. The B cell response and antibody production were largely unimpeded. Pro- and anti-inflammatory mechanisms were detected with no obvious imbalance. Concomitant to IL-6 the anti-inflammatory cytokines IL-10 and GDF-15 levels were also elevated throughout ICU treatment. T and B cell counts recovered after 8–14 days. This coincides with an overall increase of total and virus-specific IgG antibody levels, whereas anti-SARS-CoV-2-Spike-RBD IgM decreased and anti-SARS-CoV-2-Spike-RBD IgA remained relatively unaltered after ICU admission (**Figures 5B, C**). These findings went along with clinical recovery (**Figure Supp 2**). Of all patients, 69.2% survived ICU care, which compares favorably with previous cohorts of such severely sick COVID-19 patients.

Innate antiviral immune sensing *via* types I and III IFN leads to the production of pro-inflammatory cytokines (*i.e.* IL-6) within the first week after virus infection. IL-6 activates and potentiates the adaptive immune response, promoting optimal T cell regulation (29), whereas excessive levels of IL-6 can block lymphopoiesis (30) and induce lymphocyte death (31). Multiple studies suggest an association of lymphocytopenia with COVID-19 severity and mortality (32). Considering a median COVID-19 symptom onset 11 days prior to ICU admission we were unlikely to obtain information on the innate immune response within the early phase of SARS-CoV-2 infection. However, lymphocytopenia was striking in our study. All distinct lymphocyte subsets (NK cells, B cells, and T cells) exhibited low counts on admission. Most pronounced alterations related to NK cells and CD8+ T cells showing low absolute and relative numbers, which indicated an impaired or delayed cytotoxic reaction. Significantly decreased levels of *γδ* T cells were also observed. These cells combine features of innate and adaptive immunity and show antiviral activity against multiple viruses (33). Exhaustion of NK cells and CD8+ T cells has been previously described in COVID-19 and was associated with reduced CD107a degranulation and granzyme B production (34). NK cells are key players in the first line of virus defense. Their cytokine signature is comprised out of TNFα and IFN*γ*, whereas T and B lymphocytes are the prime responder to IL-1 family cytokines. IL-1*β* and IFN*γ* were well below their reference range in our patients. CXCL10 on the contrary was significantly increased. CXCL10 indicates recent IFN*γ* biological activity for up to 14 days. It is produced by monocytes and macrophages and has been suggested as a biomarker of severity in COVID-19 (35). CXCL10 elevation in our patients could be explained by increased Th1 cell activity prior to ICU admission, as well as on-going stimulation of T cell tissue migration. Other pro-inflammatory cytokines (IL-2, IL-17a) involved in the innate immune response, as well as T and NK cell proliferation, displayed low serum levels. Levels of TNFα were low during ICU treatment, which indicates that the overshooting immune response is distinct from a classical cytokine storm where TNFα is both a lead cytokine and a therapeutic target. In Chinese patients, TNFα and IFN*γ* were also not elevated in mostly mild COVID-19 (3). These findings emphasize an impaired or delayed T cell response, which is in line with data showing functionally exhausted T cells and increased PD-1 and Tim-3 expression (36).

Furthermore, naïve T helper cells were reduced in our patients. This is contrary to a single time-point analysis in Wuhan, which found increased numbers. However, the authors did not specify the time between sample collection and disease onset, and they investigated a different and diverse patient population (37). Interestingly, combined primary immunodeficiency (CVID) can be associated with a similar reduction in naïve T helper cells. In this context, naïve T helper cells <15% are associated with more severe disease, opportunistic infections and T cell-dysfunction (22). The significance of this finding remains unclear but it emphasizes reduced T cell function in patients with severe COVID-19 ARDS.

Although the absolute number of B cells was at the lower end of the reference range, SARS-CoV-2 antibody production was already detectable at the beginning of ICU therapy. Anti-SARS-CoV-2-Spike-RBD antibodies are considered to be neutralizing and present in most of COVID-19 patients nine days after onset of symptoms (38). Slightly increasing titers of anti-SARS-CoV-2-Spike-RBD IgG and concomitantly decreasing levels of anti-SARS-CoV-2-Spike-RBD IgM likely indicate ongoing B cell class switch and seroconversion. Overall, we observed a largely unimpeded response of the B cell system. Total numbers of B cells increased during ICU treatment, while absolute and relative numbers of transitional B cells decreased. Absolute numbers of naïve B cells were below their reference range. This indicated a high turnover of immature and undifferentiated cells during the lymphocytic recovery. Accordingly, post-switch memory B cells increased over time and circulating plasma blasts were elevated, resulting in rising total IgG levels. Antibody production was fully pronounced after 5–8 days in intensive care. Nevertheless, double negative B cells were significantly elevated. The exact role of these cells remains undefined. Double negative B cells have been implicated as late memory or exhausted cells in the elderly (39). CD21^low^ B cells also mark an exhausted B cell system in various conditions (40, 41); our patients again had increased percentages of this cell type. In summary, the observed alterations within the B cell subsets are in line with other studies (32) and our findings demonstrate a robust B cell response against SARS-CoV-2, which was not necessarily expected in this severely sick ICU cohort.

Maintaining balance between pro- and anti-inflammatory mechanisms is likely as important as a robust immune response. Cytokines are essential in initiating and augmenting the innate and adaptive immune response (42). Concomitant hyperinflammation has been linked to severe pulmonary dysfunction (43). In the current study IL-6 was massively increased. The dynamics of anti-inflammatory IL-10 were similar to IL-6, whereas IFN*γ*, TNFα, IL-1*β*, IL-2, IL-7, IL-17a and GM-CSF mostly exhibited levels below their respective reference range. As only two samples of the cytokine analysis were potentially affected by hemadsorption therapy, these findings suggest the absence of a cytokine storm. Seven patients (53.8%) received low dose hydrocortisone due to vasodilatory shock, and one patient was treated with methylprednisolone during ICU care. This also might have helped to prevent cytokine storm as preliminary results of the on-going RECOVERY-trial suggest decreased COVID-19 mortality (44). Moreover, GDF-15 levels were increased throughout ICU treatment. GDF-15 is a member of the anti-inflammatory TGF-*β* superfamily. While it has been shown to reduce clearance of human rhinovirus and to enhance the risk for virus-induced lung inflammation in mice (11), it counteracts inflammation (8), increases the ability of tissues to tolerate inflammatory damage *via* metabolic adaptation (9), and reduces tissue infiltration of immune cells (8). We thus hypothesize that induction of GDF-15 is an adaptation to restore a disturbed balance between pro- and anti-inflammatory cytokines. We did not measure anti-inflammatory IL-4, the signature cytokine of Th2 responses (45). However, previous studies did not demonstrate a significant alteration of this cytokine in COVID-19 (3, 46).

**Figure 8:**
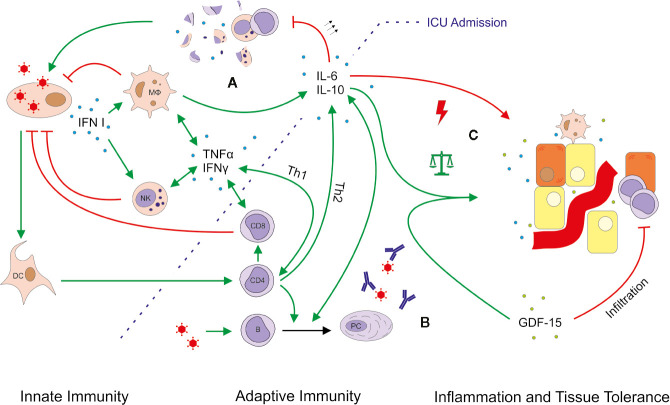
Simplified outline of immune responses in severe COVID-19 ARDS. Viral infection leads to the production of type I interferon (IFN), which activates macrophages (M*Ф*) and natural killer (NK) cells. Considering a median COVID-19 symptom onset 11 days prior to intensive care unit (ICU) admission, this first-line of defense is likely not depicted by our data. Various mediator cytokines (blue dots) including tumor necrosis factor alpha (TNFα), IFN*γ*, interleukin (IL)-6 and IL-10 connect innate and adaptive immunity and help to control the pro- and anti-inflammatory response. Severe COVID-19-induced ARDS was accompanied by massively upregulated IL-6, which might explain the observed lymphocytopenia. Excessive levels of IL-6 can block lymphopoiesis (30) and induce lymphocyte death (31). In particular NK cells and CD8+ T cells showed low absolute and relative numbers indicating an impaired or delayed cytotoxic reaction **(A)**. The B cell response and antibody production seemed largely unimpeded **(B)**. Moreover, no obvious imbalance between pro- and anti-inflammatory mechanisms was detected, as IL-10 and growth differentiation factor 15 (GDF-15, green dots) were also upregulated **(C)**. GDF-15 is a member of the anti-inflammatory TGF-*β* superfamily, which increases the ability of tissues to tolerate inflammatory damage. We thus hypothesize that induction of GDF-15 is an adaptation to restore a disturbed balance between pro- and anti-inflammatory mechanisms. Production, promotion, and positive feedback are illustrated with a green arrow (↑); inhibition and attack is depicted in red (T). The green arrows with the balanced scale represent tissue protection; the red arrow with a lightning symbol depicts inflammation. DC, dendritic cell; PC, plasma cell.

In conclusion, our study provides extended insight into the cellular and humoral immune response during severe COVID-19-induced ARDS, including changes during the recovery period (**Figure 8**). Our study has several limitations. All patients admitted to our ICU during the study period suffered from COVID-19-induced ARDS. Matched-pairs “Non-COVID control patients” were not available, and we are limited to comparisons of reference values from HC. Our study includes a small sample size and missing sampling during the initial disease phase, which was inevitable as all patients were referred to our tertiary care center from other hospitals. Neutrophil-to-Lymphocyte Ratio (NLR) trajectory data were not calculated due to the limited number of patients, as the predictive power and validity of the NLR would likely be inadequate. Flow cytometry was conducted as extended immunological diagnostics during routine clinical care. As such, our data do not provide a complete analysis of CD4+ T cell subsets and other lymphocyte subpopulations. Routine flow cytometry did not include the analysis of CD3+ CD4− CD8− double negative cells. These cells have been implicated in immune regulation of autoimmunity and inflammation. We cannot exclude the possibility of a proliferative response of these cells. However, CD3+ CD4− CD8− cells are a major producer of IL-17a, which exhibited low levels in our patients. Data on extravascular lung water (EVLW) or pulmonary vascular permeability index (PVPI) were only recorded in four patients of our study cohort. However, time-points of EVLW and PVPI measurements vary between these patients and a continuous recording of both values was not conducted in any of the patients. Thus, we cannot offer valid data on the extent of lung water. Furthermore, moderately elevated levels of GDF-15 (usually below 2 ng/ml) can be found in obesity (47), aging (48), autoimmune comorbidities (49) or cardiovascular disease (50). Levels similar to our COVID-19 patients are limited to conditions like pregnancy (51), diabetic nephropathy (52) and advanced cancers (53). After adjusting for three cases with a history of malignancy, the remaining COVID-19 patients still had median GDF-15 levels of 12.4 ng/ml. We therefore concluded that it is unlikely that the observed GDF-15 levels were biased by comorbidities. Internal HC were younger and could not be age-matched. Hence, we cannot exclude effects of immunosenescence within the comparisons of the study group and HC. However, the biggest changes in relative and absolute lymphocyte subsets occur during infancy, adolescence and in elderly patients >70 years (24, 54). As the oldest COVID-19 patient in our study was 71 years of age, effects of immunosenescence should not have critically altered the comparability. Moreover, we cannot exclude the presence of confounding variables, such as nosocomial infections, antibiotic treatment, and renal replacement therapy. However, as these confounders are not uncommon during routine ICU care, the pattern of immune response triggered by severe COVID-19 remains remarkably distinct from immunological alterations induced by other viral infections or a cytokine storm.

## Data Availability Statement

The raw data supporting the conclusions of this article will be made available by the authors, without undue reservation.

## Ethics Statement

Ethical review and approval was not required for the study on human participants in accordance with the local legislation and institutional requirements. Written informed consent for participation was not required for this study in accordance with the national legislation and the institutional requirements.

## Author Contributions

QN: Conceptualization, data curation, formal analysis, investigation, project administration, visualization, writing—original draft, writing—review and editing. MSc: Conceptualization, data curation, investigation, resources. FW, TS, MG, JH, LS, BS, and MSi: Investigation. DW, NS: Resources. PK: Supervision. JW: Investigation, resources, writing—review and editing. PM: Funding acquisition, resources, supervision, writing—review and editing. CL: Conceptualization, data curation, investigation, supervision, writing—original draft, writing—review and editing. All authors contributed to the article and approved the submitted version.

## Conflict of Interest

MSc reports grants and personal fees from Chugai/Roche, personal fees from Hexal Sandoz, personal fees from AbbVie, personal fees from Novartis, personal fees from Janssen-Cilag, grants and personal fees from BMS, personal fees from Boehringer/Ingelheim, personal fees from Gilead, outside the submitted work. MG reports travel grants from AbbVie, travel grants from Roche/Chugai, travel grants from Hexal, travel grants from Eli Lilly, outside the submitted work. JW reports personal fees from CatalYm GmbH, outside the submitted work.

The remaining authors declare that the research was conducted in the absence of any commercial or financial relationships that could be construed as a potential conflict of interest.
