# Supplementary material for: Pro- and Anti-Inflammatory Responses in Severe COVID-19-Induced Acute Respiratory Distress Syndrome—An Observational Pilot Study
Source: Front Immunol. 2020 Oct 6;11:581338. doi: 10.3389/fimmu.2020.581338 (PMC7573122; doi:10.3389/fimmu.2020.581338)
Supplement: Supplementary file 1 [file DataSheet_1.docx]

Supplementary Material

# Supplementary Figures and Tables

Table Supp1: Short and long-term use of immunomodulatory treatment in the study population.

| **Immunomodulation** | **No. patients** |
| --- | --- |
| Long-term use of immunomodulatory medication |  |
| Prednisolone | 2 |
| Rituximab^†^ | 2 |
| Sarilumab | 1 |
| Leflunomid | 1 |
| Amphotericin B | 1 |
| Immunomodulatory treatment during intensive care |  |
| Immunoglobulin^†^ | 3 |
| Plasma of convalescent patients^†^ | 1 |
| Tocilizumab^†^ | 1 |
| Hydrocortisone (low dose 200 mg/day)^†^ | 7 |
| Methylprednisolone (5 mg/day) | 1 |
| G-CSF^†^ | 1 |
| Renal replacement therapy with hemadsorption | 8 |

One patient is highlighted with a footnote symbol (†), as he received a multitude of different therapies. G-CSF, granulocyte colony-stimulating factor; No., number of patients.

##
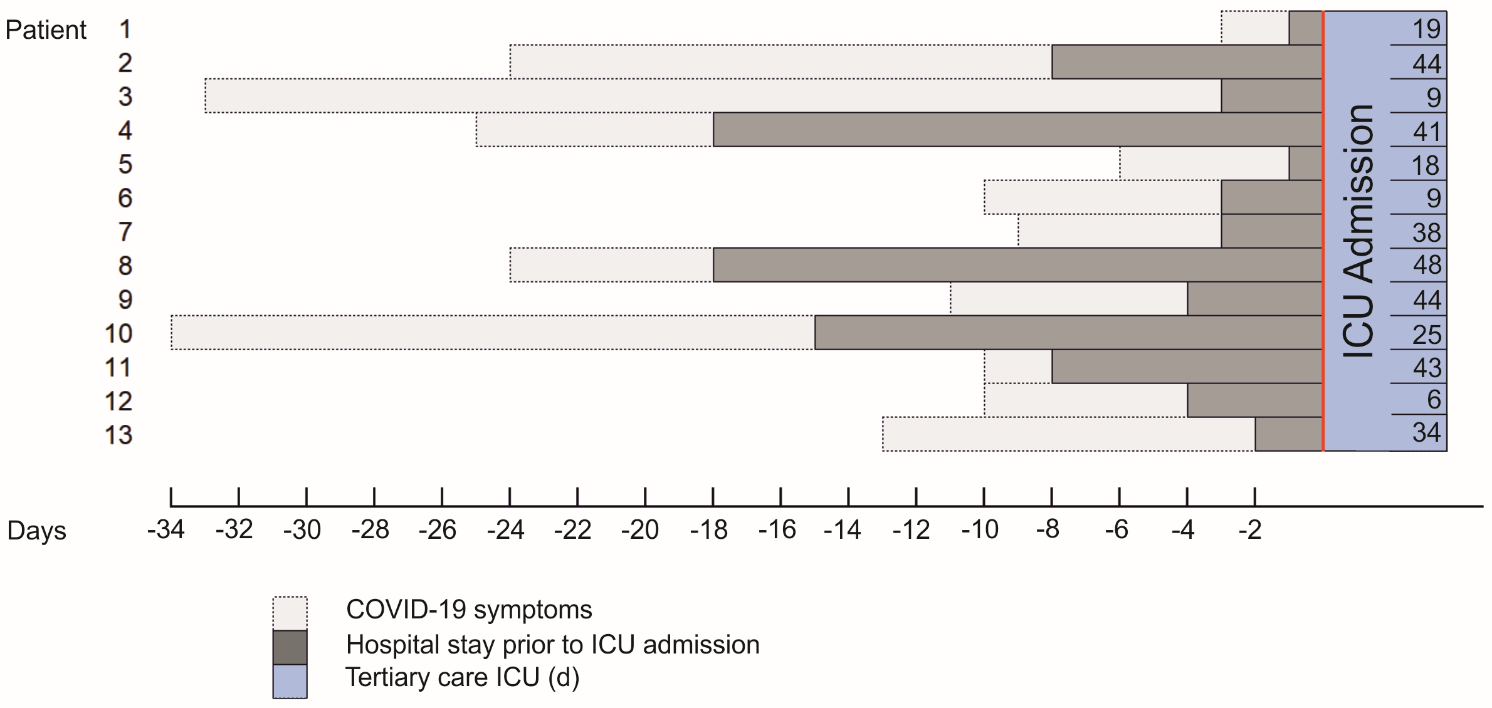


**Figure Supp1:** Clinical course of each patient within the current study population. The horizontal bars start with COVID-19 symptom onset (light gray bars), followed by hospital admission (gray bars). The red line marks intensive care unit (ICU) admission. Numbers displayed on the right denote the number of days in intensive care.


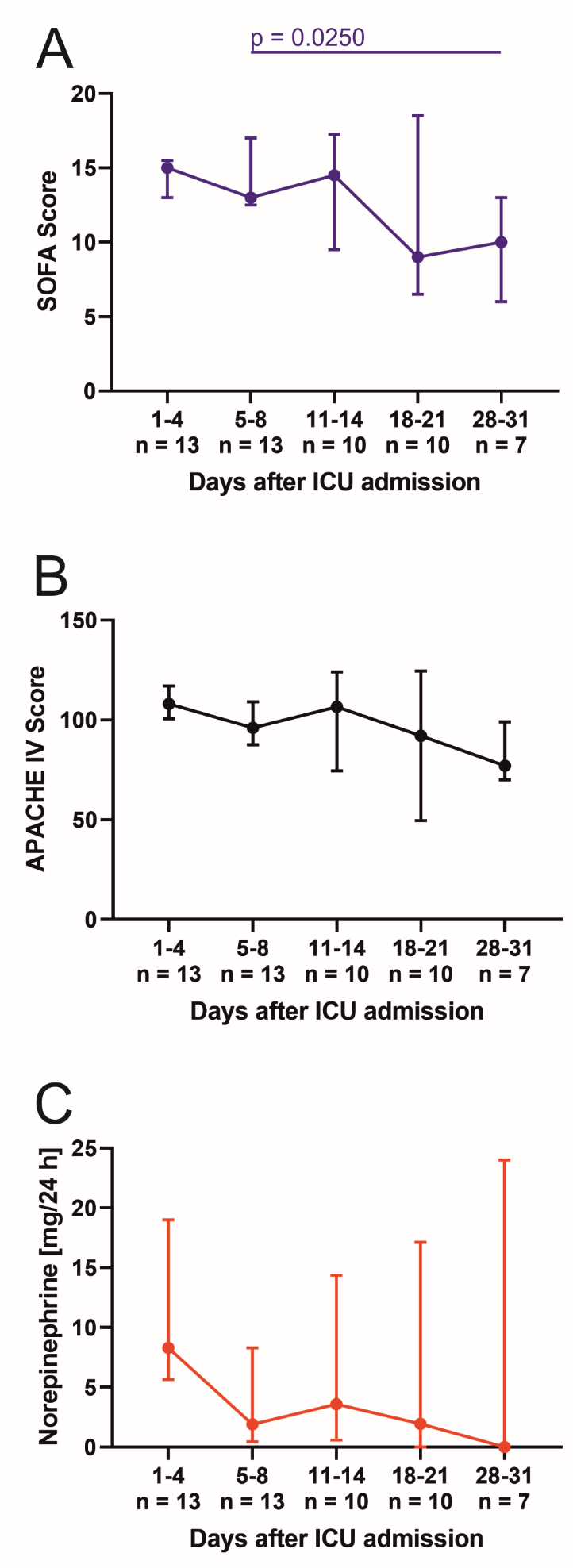
**Figure Supp2:** Selected parameters showcasing severity of disease and mortality risk of the current study population. Sequential organ failure assessment (SOFA) score and acute physiology and chronic health evaluation (APACHE) IV score remained high throughout days 11 - 14 **(A - B).** After this time-point both scores dropped. Simultaneously, the doses of norepinephrine, required for circulatory support, declined **(C)**. Hence, clinical recovery went alongside recovery of T and B cell responses as well as fully pronounced SARS-CoV-2 antibody production.
